# Nobloedischia rasnitsyni, a new genus and species of Oedischiidae (Orthoptera) from the Lower Permian Wellington Formation of Oklahoma, USA

**DOI:** 10.3897/zookeys.130.1327

**Published:** 2011-09-24

**Authors:** Roy J. Beckemeyer

**Affiliations:** 1 Division of Entomology (Paleoentomology), Natural History Museum, and Department of Ecology & Evolutionary Biology, 1501 Crestline Drive – Suite 140, University of Kansas, Lawrence, Kansas 66049-2811 USA; Correspondence address: 957 Perry Ave., Wichita, KS 67203-3141, USA

**Keywords:** Nobloedischia, fossil insect, Noble County, paleoentomology

## Abstract

*Nobloedischia rasnitsyni* **gen. et sp. n.** (Oedischiidae) is described from the Lower Permian Wellington Formation of Noble County, Oklahoma. The genus is similar to both *Petrelcana* (Oedischiidae: Mezenoedischiinae) and *Oedischia* (Oedischiidae: Oedischiinae) and is left unplaced at the subfamily level. The new species is the twelfth Orthoptera species and the fourth species of Oedischiidae from these deposits.

## Introduction

The Oedischiidae are considered to be the most basal family of orthopterans; they occurred from the Late Carboniferous through the Late Permian (Sharov 1971; Gorochov 1987, 1995; Gorochov and Rasnitsyn 2002). For the most part, Oedischiidae are known only be their forewings; in the Carboniferous (Commentry, France) *Oedischia* Brongniart, 1885, the hind legs were saltatorial (Sharov 1971), and in the Lower Permian *Uraloedischia* Sharov, 1968 (Urals, Russia), prehensile spines on the legs and pointed denticles on the mandibles suggested oedischiids were predacious (Sharov 1971; Gorochov and Rasnitsyn 2002). The wing form and venation suggest they were active fliers (Sharov 1971). Besides *Oedischia*, a second Carboniferous genus, *Sinoedischia* Hong, 1985, is known from the Shanxi of China (Gorochov and Rasnitsyn 2002; Meng et al. 2006).

Oedischiids became more diverse in the Permian; known genera include *Afroedischia* Geertsema & van Dijk, 1999 (Middle–Upper Permian, South Africa. The Laingsburg Formation was dated as Lower Permian by Geertsema and van Dijk 1999, but is now dated as Middle–Upper Permian by Fildani et al. 2009.), *Iasvia* Zalessky, 1934 (Lower Permian, Russia, and Middle Permian, France; Béthoux et al. 2002), *Macroedischia* Sharov, 1968, *Tettoedischia* Sharov, 1968, and *Uraloedischia* Sharov, 1968 from the Lower Permian (Kungurian) of Russia, *Metoedischia* Martynov, 1928, *Elcanoedischia* Gorochov, 1987, and *Mezenoedischia* Gorochov, 1987 from the Middle Permian (Kazanian) of Russia, and, from the Lower Permian Wellington Formation of the United States, in subfamily Elcanoedischiinae Gorochov, 1987: *Kansasoedischia* Gorochov, 1987 (Kansas), and in Mezenoedischiinae Gorochov, 1987: *Petrelcana* Carpenter, 1966 (Kansas), and *Pseudoiasvia* Bethoux & Beckemeyer, 2007 (Oklahoma).

Of the more than 200 species of insects known from the Lower Permian Wellington Formation of Kansas and Oklahoma, USA (Beckemeyer and Hall 2007), eleven are in the order Orthoptera: eight from the Elmo, Kansas deposits (Tillyard 1932; 1937; Carpenter 1943, 1966; Gorochov 1987), and three from the Midco, Oklahoma (Noble County) formations. Four of the eleven species, one from Kansas and three from Oklahoma, were described only recently (Béthoux and Beckemeyer 2007; Beckemeyer 2011). The new taxon described here constitutes the tenth genus and twelfth species of Wellington Formation Orthoptera (see Beckemeyer 2011 for a list of previously described species). It seems most closely related to *Oedischia* and *Petrelcana*.

## Materials and methods

**Figures 1–3.**
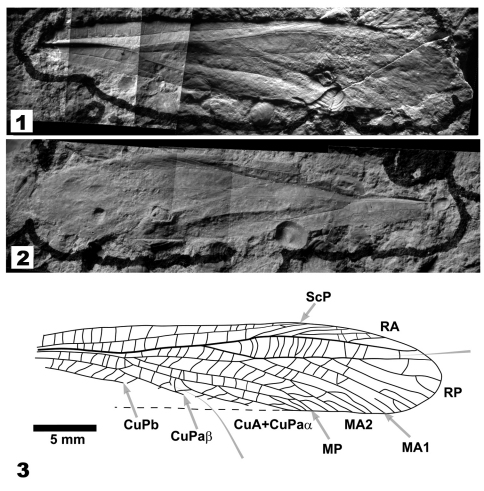
*Nobloedischia rasnitsyni* gen. et sp. n. **1.** Holotype specimen, part, OSU826a. Forewing in dorsal aspect **2.** Holotype specimen, counterpart, OSU826b. Forewing in ventral aspect **3.** Venation reconstruction and notation following Béthoux and Nel 2002. Photographs and drawing to same scale; scale bar 5 mm.

This description is based on a forewing specimen (Figs 1, 2) collected by Don Arnold and Rick Grantham of Oklahoma State University (OSU). The holotype is in the K. C. Emerson Museum, Department of Plant Pathology and Entomology, OSU. The specimen comprises part and counterpart of a nearly complete wing, absent approximately the basal 20% of the wing length (including the precostal area), the anal field, and the posterior margin of the cubital field; cross veins are poorly preserved in the distal radial and medial fields.

Photomicrographs of the part and counterpart were made using a Nikon 990 digital camera with an American Optical dissecting microscope and an external strobe flash (Nikon SB-26). Flash orientation was optimized to show details of interest. The photographed images were processed using Adobe Photoshop 6.0 and imported into a vector-graphics software program (XARA Extreme 4.0, XARA Group, Ltd., London), where they were assembled into composite images. The venation reconstruction drawing was made as an overlay (Fig. 3).

Venation interpretation and notation follow the system of (Béthoux and Nel (2001, 2002). Since that system is relatively new, not uniformly accepted, and not in as wide use as the terminology of Sharov (1971), (Gorochov (1987, 1995), Carpenter (1992), and Gorochov and Rasnitsyn (2002), I list here the relationship between the most common notation systems:

**Table:** 

Béthoux and Nel	Sharov/ Gorochov	Carpenter	Gorochov and Rasnitsyn
ScP	Sc	SC	SC
RA	RA	R	R_1_
RP	RS	RS	RS
MA1	1MA_1_	MA1	MA_1_
MA2	2MA_1_	MA2	MA_2_
MP	MA_2_	MP	MP
CuA+CuPaα	MP+CuA_1_	CuA	M_5_+CuA_1_
CuPaβ	CuA_2_	CuA	CuA_2_
CuPb	CuP	CuP	CuP

## Systematic paleontology

**Order Orthoptera Olivier, 1789**

**Family Oedischiidae Handlirsch, 1906**

### Nobloedischia

Beckemeyer gen. n.

urn:lsid:zoobank.org:act:5B75F078-99C6-457E-AFB6-CBE9364C2328

http://species-id.net/wiki/Nobloedischia

#### Composition.

Type species *Nobloedischia rasnitsyni* Beckemeyer, sp. n.

#### Etymology.

A combination of Noble County and the family name Oedischiidae.

#### Diagnosis.

*Nobloedischia* is differentiated from *Oedischia* by branches of RA directed distally, first branching of RA at level of fusion of MA1 and RP; fusion of RP and MA1 (the anterior branch of MA) long rather than limited to a point of contact; field between MP and CuA+CuPaα narrower than area between MP and MA/MA2 at least in basal 2/3 of length; CuA+CuPaα and its branches reach posterior wing margin obliquely.

Differs from *Petrelcana* by width of costal and subcostal fields at level of branching of M about equal rather than costal field much wider than subcostal field; cross veins between RA and RP without secondary cross veins; branching of MA moderately remote from anastamosis with RP (as in Gorochov 1995, Fig. 142) rather than MA branching close to fusion with RP, stem of MA1 before fusion an obvious branch of the longitudinal vein rather than this segment of MA1 very short and appearing as a thickened cross vein between MA/MA2 and RP+MA1 (as in Gorochov 1995, Fig. 144; stems of CuA and CuPaα about equal rather than stem of CuA very short.

### Nobloedischia rasnitsyni

Beckemeyer sp. n.

urn:lsid:zoobank.org:act:01F11D63-3DBD-4F2E-9F39-C0B499755E97

http://species-id.net/wiki/Nobloedischia_rasnitsyni

Figs 1–3

#### Type locality.

Noble County, Oklahoma, USA; Wellington Formation, Artinskian, Lower Permian.

#### Type material.

**Holotype:** Oklahoma State Museum Fossil Insect Specimen No. 826a, part (Fig. 1) and 826b, counterpart (Fig. 2), comprising the only Orthoptera fossil on slabs densely covered with multiple insect wing fragments of varied orders and with Conchostraca fossils as well. The slab containing 826a is approximately 12 by 21 cm and contains a total of 88 insect specimens (818a through 905a); the second slab (8 by 17 cm) contains 78 insect specimens (818b through 896b).

#### Etymology.

The specific epithet, *rasnitsyni*, is an honorific for eminent scientist Dr. Alexandr P. Rasnitsyn in recognition of his long and productive career, and his invaluable and varied contributions to paleoentomology.

#### Diagnosis.

As for genus.

#### Description.

Forewing. Preserved length 32 mm, estimated length 39 mm (basal 20%, including precostal area, missing); width 7 mm. ScP terminates at 78% wing length, cross veins simple, moderately spaced, and oriented normal to costal margin and ScP, maximum width of costal field 1.4 mm, maximum width of subcostal field 1.0 mm, ratio width of subcostal to width of costal field 1.4:1. RA sigmoidal in form, bending anteriorly at level of separation of Cu and M (35% of wing length), bending posteriorly at level of fusion of MA and RP, where RA branches; RA branching anteriorly pectinate, six branches reaching wing margin, basal branches intersecting margin obliquely, distal branches nearly longitudinal in orientation; posterior–most branch of RA terminating at 95% wing length; cross veins between ScP and RA similar in form and spacing to those between ScP and costal margin. RP origin at 51% wing length; RP fusion with MA1 at 62% of wing length, length of fusion 0.9 mm; RP with four posteriorly pectinate branches, simple or distally twigged, first branch at 72% wing length, anterior–most branch straight and terminating near apex of wing at 98% wing length; cross veins between RA and RP with veins simple, some bowed with center of curvature basal. M+CuA branches at 35%, M branches at 37%, length of M from separation from M+CuA to branch 0.9 mm, MA branches at 59%, MA2 branches at 69% of wing length each branch with terminal twig, MA1 apparently simple, terminating at 89%, free length of MA1 before fusion with RP 1.0 mm. MP distally twigged, terminating on posterior margin at 74% and 77% wing length. CuA+CuPaα with four branches; the basal branches of CuA+CuPaα intersect the posterior margin obliquely, with the angle between the branches and the wing margin increasing distally (angles of intersection are 12°, 13°, 23°, and 32°, for the successive branches); CuPa branches at 34% of the wing length, free length of CuPaα before fusion with CuA 1.1 mm, length of CuA from separation from M+CuA to fusion with CuPaα 0.9 mm, CuPaβ straight as preserved, cross veins between CuPaβ and CuA+CuPaα normal to the longitudinal veins, becoming oblique and oriented longitudinally after the first branching of CuA+CuPaα; short segment of CuPb that is preserved (extending from 20% to 35% of wing length) is straight; anal veins not preserved.

## Discussion

The combination of character states of *Nobloedischia rasnitsyni* make placement in one of Gorochov’s subfamilies somewhat problematic; it would perhaps come closest to fitting in Oedischiinae. *Nobloedischia* shares with *Oedischia* the characters: origin of anterior branch of MA moderately remote from its anastamosis with RP (as in Gorochov 1995: Fig. 142); distance from branching of M+CuA to branching of M subequal to distance from branching of M+CuA to fusion of CuA and CuPaα, and greater than length of cross veins between CuA+CuPaα and MP; ratio of width of costal field (anterior margin–ScP) to width of subcostal field (ScP–RA) less than 1.5; field between RA and RP with some cross veins bowed and/or sigmoidal rather than straight; CuPaα medium sized basal of its fusion with CuA (last three characters were used by Béthoux and Nel 2002, to distinguish the clade (*Gerarus bruesi* Meunier, 1909 + *Oedischia williamsoni*)). On the other hand, *Nobloedischia* is differentiated from *Oedischia* by the characters listed in the Diagnosis (vide supra). Béthoux and Nel (2002) used a total of 74 characters in their cladistic phylogenetic analysis of the Orthoptera; I have not repeated that analysis, but did code the characters and found that of the 61 character states that could be compared (the remaining characters were not preserved in available specimens of one or the other of the taxa) between *Nobloedischia* and *Oedischia*, 52 matched and 9 did not.

For the other oedischiids, the next closest pairing was between *Nobloedischia* and *Elcanoedischia*, with 45 of 58 character states matching. However, *Nobloedischia* differs from genera in Elcanoedischiinae Gorochov 1987 by branching of MA moderately remote from anastamosis with RP (as in Gorochov 1995: Fig. 142) rather than MA branching close to fusion with RP, with this short segment of MA appearing as a thick cross vein between MA/MA2 and RP+MA1 (as in Gorochov 1995: Fig. 144). *Nobloedischia* can be differentiated from genera in Tettoedischiinae Gorochov, 1987 by costal field not greatly wider than subcostal field; base of MP well basal rather than at or distal to base of RP; branching of CuPb basal rather than distal to branching of M+CuA. *Nobloedischia* differs from genera in Mezenoedischiinae by the features listed in the Diagnosis section for *Petrelcana* (vide supra). Because of the presence of these conflicting character states, at this point I prefer to leave the subfamilial assignment undetermined.

The high number of densely distributed specimens (wings and wing fragments) present on the slabs containing this species seems to support Hall’s (2004) thesis that the Oklahoma Wellington Formation insect deposit facies are derived from marginal marine lagoon sediments, with insect remains likely comprised of allochthonous material washed in by streams.

## Supplementary Material

XML Treatment for Nobloedischia

XML Treatment for Nobloedischia rasnitsyni
